# New respiratory syncytial virus immunization products in low- and middle-income countries: potential for cost-effective impact on a high burden of disease in young infants

**DOI:** 10.1186/s12916-023-02883-x

**Published:** 2023-05-15

**Authors:** Padmini Srikantiah, Keith P. Klugman

**Affiliations:** Pneumonia Program, Global Health Division, Bill & Melinda Gates Foundation, 500 5th Ave North, Seattle, WA 98109 USA

**Keywords:** Respiratory syncytial virus, Maternal vaccine, Infant monoclonal antibody, Burden, Cost effectiveness, Low- and middle-income countries

## Abstract

As new, efficacious respiratory syncytial virus (RSV) immunization products reach the market, affordable pricing as well as improved estimation of disease burden and the full potential and cost effectiveness of RSV prevention in the hardest hit geographies in low- and middle-income countries are critical to inform country adoption and enable maximum impact against infant disease and mortality globally. The data reported in the special issue underscore the enormous burden, and associated cost, of RSV disease in young infants in several LMICs, including Kenya and South Africa, as well as the potential for RSV maternal vaccines or long-acting monoclonal antibodies, to be cost-effective and possibly even cost-saving.

## Background

Respiratory syncytial virus (RSV) is the most common cause of severe lower respiratory tract infection in the first 6 months of life where the overwhelming burden of death occurs mostly in low- and middle-income countries (LMICs) [1]. After a decades-long gap in the development of successful RSV prevention products, positive pivotal phase 3 trial results were reported in 2022 for both an RSV maternal vaccine [2] and a long-acting single-dose monoclonal antibody (mAb) [3, 4], signaling the possibility of a new era in RSV prevention in young infants. Once these products are licensed in high-income settings, it will be critical to ensure timely and affordable access to them in LMICs to maximize their impact on global RSV burden and mortality in young infants. It is equally important to generate relevant data and tools to guide country-level policy on introduction of RSV maternal vaccines and mAbs in these geographies, where the public health value of RSV prevention might be under-recognized, and decision makers are frequently tasked with prioritizing among multiple competing public health challenges. The papers in the current supplement report country-specific estimates of infant RSV disease burden and cost of illness in several LMICs as well as analyses that provide insight into the cost effectiveness of RSV maternal vaccines and mAbs that will be critical to support informed policy decisions.

## Main text

Investigators in Kenya and South Africa took advantage of data from existing sentinel acute respiratory illness surveillance platforms combined with healthcare utilization survey or local population census data to extrapolate and estimate RSV burden of disease at national and sub-national levels (Nyawanda, et al. https://doi.org/10.1186/s12916-023-02787-w and Moyes et al., https://doi.org/10.1186/s12916-023-02854-2). In both settings, the data reveal an enormous burden of RSV-associated mild and severe respiratory illness in young infants and show that illness detected in the healthcare setting represents only a part of the full RSV disease burden. While 54% of severe RSV disease in children under 5 years was medically attended in South Africa, non-medically attended illnesses accounted for a majority of the RSV burden in Kenya. Among infants less than 6 months of age in Kenya, the rate of non-hospitalized severe RSV illness was three times that of hospitalized disease (6404/100,000 infants vs. 2075/100,000 infants), while 60% of annual RSV-associated deaths were estimated to occur outside the hospital. These findings corroborate recently published data from community mortality studies conducted in several LMICs that showed a high unmeasured burden of RSV mortality outside of the healthcare settings among infants less than 6 months of age [5]. Indeed, in some low-income countries, the proportion of all infant deaths that occur in the community may be as high as 80% [1, 6].

Notably, the investigators in both Kenya and South Africa provided disease burden estimates in narrow age strata, including month-by month age bands for the first year of life, that reveal the highest incidence of disease and risk of RSV-associated death is, as expected, in the first 2 months of life. In South Africa, the rate of severe RSV disease was 14,736 per 100,000 infants in the first month of life, compared to 2,156 per 100,000 in the eleventh month of life (Moyes et al.). In Kenya, where non-hospitalized SARI accounted for a greater proportion of overall cases, the rate of severe RSV disease in the first month of life (10,553 per 100,000 infants) was more than three times greater than that estimated for the sixth month of life (3246 per 100,000 infants). These granular burden data provide critical insights into the potential impact of RSV maternal vaccines or mAbs, which have demonstrated highest efficacy in the first few months of life [2]. The burden analyses reported also highlight the utility of including testing for RSV in sentinel pediatric severe acute respiratory illness (SARI) surveillance platforms in LMICs [7]. Even in the absence of robust population-based denominators, systematically collected country-specific surveillance data can be used to highlight the importance of RSV among hospitalized children and help national policymakers to recognize the enormous healthcare system burden that RSV poses.

The investigators in Kenya and South Africa further assessed the direct and indirect costs of RSV illness incurred on the household and healthcare system in their respective geographies. In Kenya, where the focus was on the cost of taking care of a child hospitalized with severe RSV lower respiratory tract infection (LRTI), the total mean cost to both health system and household ranged from USD 119 per episode in Kilifi District, where the KEMRI-Wellcome Trust Programme (KWTRP) provides significant external financial support for delivery of pediatric medical services, to USD 527 in Siaya District, which relies on user fees and national government support, and might be more representative of other regions of Kenya (Nyiro et al.) In South Africa, investigators utilized their RSV burden data analysis to further estimate the cost of RSV illness in children < 5 years at the national level to be greater than USD $137 million. Stratification of these costs by granular age bands revealed that the greatest financial burden of RSV illness is in the first 6 months of life, with the highest cost associated with the first 2 months of life—again underscoring the potential benefit of maternal vaccines and mAbs that demonstrate greatest impact in this age group. Notably, these cost of illness analyses focus on the acute RSV LRTI episode but do not take into account potential costs associated with recurrent LRTI or wheeze later in childhood, both of which have been associated with severe RSV disease early in life [8].

Severe RSV illness was associated with overwhelming out-of-pocket personal expenses in South Africa, averaging > 100% of mean monthly income. This was also observed in Kenya, where more than 90% of households reported that their family finances were adversely affected by the costs of this RSV LRTI episode. Although Vietnam represents a different socio-economic geography, a cost of illness study conducted there indicated that, even in the presence of heavy government subsidies for inpatient medical care, out of pocket expenditure for an RSV LRTI hospitalization ranged from 55 to 110% of the monthly per-person minimum wage in Ho Chi Minh City (Do et al. https://doi.org/10.1186/s12879-023-08024-2). A systematic review of published RSV cost of illness studies reveals an overall paucity of data from LMICs, but similarly indicates high out-of-pocket costs to the family in Bangladesh and Malawi, 24% and 32% of monthly household income, respectively (Wittenauer et al. https://doi.org/10.1186/s12916-023-02792-z). The staggering household level financial burden of illness observed in LMICs might have broader repercussions for family health and welfare and deserves more discussion and attention in policy discussions.

Based on the refined disease burden and cost of illness data in Kenya and South Africa, Koltai et al. have estimated the cost effectiveness (CE) of an RSV maternal vaccine and an RSV long-acting infant mAb in these countries (Koltai et al. https://doi.org/10.1186/s12916-023-02806-w). Utilizing the reported RSVPreF maternal vaccine phase 3 efficacy (81.8% against severe RSV disease at 90 days) [2] and assuming a dose price of $USD 5, the analysis showed a favorable incremental cost effectiveness ratio (ICER) of USD $180 per disability-adjusted life year (DALY) averted in Kenya. In South Africa, the same maternal vaccine, at both USD $5 and USD $10, was actually cost-saving. Notably, the investigators assumed a fixed duration of 3 months for maternal vaccine efficacy, such that protection drops to zero after 90 days. In reality, while vaccine efficacy declines after 90 days, there is some continued protection beyond this period [2], and an RSV maternal vaccine could be more cost-effective (lower ICERs) in these geographies. A similar analysis of cost effectiveness for the RSV mAb, which utilizes the reported pooled phase 2b/3 efficacy of nirsevimab (77.3% against RSV hospitalization at 150 days) [4] and a dose price of USD $10, while again cost-saving in South Africa, resulted in a higher ICER (USD $686 per DALY averted) for Kenya (Koltai et al.). These findings underscore how sensitive cost-effectiveness metrics are to changes in the price, efficacy, and duration of protection of an RSV immunization product and highlight the need for similar analyses in a diversity of LMICs. A product that confers protection against severe RSV disease for 6 months at a lower dose price (e.g., < USD $4), for instance, could have the potential to be cost-saving in more LMICs (Mahmud et al. https://doi.org/10.1186/s12916-023-02827-5), highlighting the importance of ensuring affordability of RSV maternal vaccines and mAbs. Furthermore, these CE analyses are focused on the potential immediate impact of maternal vaccines or mAbs on RSV specific disease and associated mortality. RSV immunization products might have however the potential to impact all-cause pneumonia [3, 9], which could further improve the CE of these vaccines and mAbs. Generating data on the impact of maternal vaccines and/or mAbs on relevant endpoints, including RSV disease, all-cause pneumonia, and even mortality, in LMICs will be important to understand the full impact of RSV prevention.

## Conclusions

RSV is the leading cause of pneumonia and related mortality in young infants in LMICs. As new, efficacious immunization products reach the market, affordable pricing as well as improved estimation of infant disease burden and the full potential and cost effectiveness of RSV prevention in the hardest hit geographies will be critical to inform country adoption and enable maximum impact against infant disease and mortality globally.

## Data Availability

Not applicable.
